# Role of metabolically active hormones in the insulin resistance associated with short-term glucocorticoid treatment

**DOI:** 10.1186/1477-5751-5-14

**Published:** 2006-09-11

**Authors:** Jeetesh V Patel, David E Cummings, John P Girod, Alwin V Mascarenhas, Elizabeth A Hughes, Manjula Gupta, Gregory YH Lip, Sethu Reddy, Daniel J Brotman

**Affiliations:** 1 Haemostasis Thrombosis and Vascular Biology Unit, University Department of Medicine and Sandwell Medical Research Unit, Sandwell and West Birmingham Hospitals NHS Trust, West Midlands, UK; 2 Department of Medicine, University of Washington, Veterans Affairs Puget Sound Health Care System, Seattle, WA, USA; 3 Department of Cardiology, University of Pittsburgh Medical Center, Pittsburgh, PA, USA; 4 Departments of General Internal Medicine and Endocrinology, Diabetes and Metabolism, Cleveland Clinic Foundation, Cleveland, OH, USA

## Abstract

**Background:**

The mechanisms by which glucocorticoid therapy promotes obesity and insulin resistance are incompletely characterized. Modulations of the metabolically active hormones, tumour necrosis factor alpha (TNF alpha), ghrelin, leptin and adiponectin are all implicated in the development of these cardiovascular risk factors. Little is known about the effects of short-term glucocorticoid treatment on levels of these hormones.

**Research methods and procedures:**

Using a blinded, placebo-controlled approach, we randomised 25 healthy men (mean (SD) age: 24.2 (5.4) years) to 5 days of treatment with either placebo or oral dexamethasone 3 mg twice daily. Fasting plasma TNFα, ghrelin, leptin and adiponectin were measured before and after treatment.

**Results:**

Mean changes in all hormones were no different between treatment arms, despite dexamethasone-related increases in body weight, blood pressure, HDL cholesterol and insulin. Changes in calculated indices of insulin sensitivity (HOMA-S, insulin sensitivity index) were strongly related to dexamethasone treatment (*p *< 0.001).

**Discussion:**

Our data do not support a role for TNF alpha, ghrelin, leptin or adiponectin in the insulin resistance associated with short-term glucocorticoid treatment.

## Background

Glucocorticoids are common therapy for inflammatory conditions, but they generate a diverse array of unwanted side effects [1]. Their mechanisms of action involve the activation of transcription factors that interact with a battery of responsive genes, stimulating inflammatory and immuno-regulatory cross-talk [2]. Glucocorticoid therapy promotes both insulin resistance [3,4] and central obesity [5], perpetuating cardiovascular risk [6]. However, the mechanisms of glucocorticoid-mediated obesity remain incompletely characterized, and the impact of glucocorticoids on hormones and cytokines that regulate hunger, satiety and adiposity remain unclear. Therefore, we sought to determine the acute effects of glucocorticoid administration on tumor necrosis factor – alpha (TNF alpha), ghrelin, leptin, and adiponectin–all hormones and cytokines thought to play an important role in the regulation of adiposity [7].

TNF alpha represents a potential link between adiposity and insulin resistance, since circulating levels are associated with adipose mass and exogenous administration increases insulin resistance [8]. Ghrelin, an orexigenic gut peptide, is implicated in long- and short-term body weight regulation. Exogenous administration blocks insulin action, both via indirect effects on other hormones and via direct actions in the liver [9]. Leptin is an adipocyte-derived hormone that circulates in proportion to body fat stores; it promotes weight loss and increases insulin sensitivity [10]. Adiponectin, also from adipose tissue, increases insulin sensitivity and can decrease body weight [11]. We hypothesised that short-term glucocorticoid treatment among healthy individuals would cause insulin resistance, with coordinated increases in TNF alpha, leptin and ghrelin, and decreases in adiponectin. For this study, we used a synthetic glucocorticoid, dexamethasone, which selectively targets the glucocorticoid receptor and glucocorticoid responsive genes, without significant mineralocorticoid effects [1].

## Methods

Weperformed a randomized, double-blind, placebo-controlled study in healthy young men ages 19–39 who were recruited by local advertisements. The methods are described in detail elsewhere [12]. Briefly, subjects were treated with dexamethasone 3 mg twice daily for 5 days or with placebo. Fasting 8 AM blood samples were obtained before and after the intervention. Potential subjects were excluded if they had any of the following: ongoing medical or psychiatric illnesses, regular use of prescription or non-prescription medications, illicit drug use or excessive alcohol use, surgery or hospitalization in the preceding 3 months, exposure to exogenous glucocorticoids in the preceding year, or non-traditional sleep/wake habits (e.g.: night shift work, frequent travel across time zones). Subjects were advised to maintain their usual sleep-wake schedule, exercise and dietary habits during the study, and were advised not to take any prescription medications, over-the-counter medications, or alcohol during the protocol. All subjects provided written informed consent. Investigators and subjects were blinded to treatment assignment, and compliance was confirmed by measuring post-treatment cortisol levels (undetectable in all subjects who received dexamethasone). The Cleveland Clinic Foundation Institutional Review Board approved the protocol.

### Laboratory data

Separated serum and EDTA plasma were stored at -70°C for batch analysis. Serum levels of glucose, cholesterol and triglycerides were determined using routine autoanalyser assays. Insulin levels were determined using an enzyme immunoassay (AIA NexIA, Tosoh Bioscience, S. San Francisco, CA). TNFα, leptin and adiponectin levels were measured by enzyme linked immunosorbant assay (ELISA) in plasma, using commercially available antibodies (R&D Systems, Abingdon, UK). Plasma ghrelin was measured by radio-immunoassay (Phoenix Pharmaceuticals, Belmont, CA).

Insulin sensitivity was assessed using the homeostatic model (HOMA-S), which is directly related to fasting insulin and glucose levels [13]. A weighted combination of fasting insulin and triglycerides [14], 'insulin sensitivity index' (ISI), was also used as surrogate a marker of insulin sensitivity.

### Power calculation and statistical analysis

We hypothesised that dexamethasone treatment would significantly decrease HOMA-S. Based on previous data [3,14], 12 patients would be sufficient to observe a significant (p < 0.05) decrease of at least 1.9 in HOMA-S using a two-sided test at 80% power. The change from baseline to post-intervention was calculated in each variable (variables with highly skewed distributions were log-transformed prior to this). Data were analysed using parametric and non-parametric tests, with ANOVA and multiple linear and logistic regression analyses as appropriate (SPSS Inc., Chicago, IL). Partial correlation analysis (two-tailed) was used to adjust the effects of treatment arm, used for bivariate analysis among all subjects.

## Results

Of the 25 male subjects (24.2 (5.4) years), 13 were randomized to dexamethasone and 12 to placebo. Baseline plasma TNFα, ghrelin, leptin and adiponectin were comparable among subjects in the dexamethasone and placebo groups before intervention (all *P *> 0.15), and these values did not change significantly after treatment (Table 1). In contrast, there were significant increases in body-mass index (BMI), systolic blood pressure, HDL cholesterol, serum insulin and insulin resistance amongst subjects on glucocorticoid therapy compared with those on placebo (described elsewhere [12]). All subjects treated with dexamethasone had undetectable post-treatment morning cortisol levels, confirming compliance with the intervention. Mean changes (pre-treatment value minus post-treatment value) in TNFα, leptin and adiponectin were not significantly correlated with changes in cardiovascular risk factors, but there was a modest association between changes ghrelin and diastolic blood pressure (*P *= 0.04), after adjusting for treatment arm.

**Table 1:** Plasma levels of TNF alpha, ghrelin, leptin and adiponectin among healthy male volunteers during short-term intervention with either dexamethasone or placebo.

Circulating levels of metabolically active hormones	Pre-treatment		Post-treatment		Significance of the difference between changes (pre-post), Placebo vs. Dexamethasone (p-value)
		
	Placebo (n = 12)	Dexamethasone (n = 13)	Placebo (n = 12)	Dexamethasone (n = 13)	
Insulin sensitivity (HOMA-S)*	7.55 (6.885–8.85)	8.28 (7.26–9.85)	7.07 (6.03–9.96)	5.46 (4.79–7.39)	< 0.001
Insulin sensitivity index (ISI)*	1.16 (0.81–1.58)	1.35 (0.95–2.48)	0.94 (0.53–2.46)	0.38 (0.26–0.77)	< 0.001
Tumor Necrosis Factor alpha (pg/ml)	660 (230–1580)	600 (0–1080)	620 (240– 1460)	580 (0–1080)	0.68
Ghrelin (pg/ml)	422 (239–591)	342 (285–497)	359 (265–465)	291 (188–347)	0.19
Leptin (pg/ml)	14,400 (8,400–25,600)	10,700 (4,200–19,800)	17,800 (2,700–22,600)	15,900 (4,200–30,500)	0.85
Adiponectin (ng/ml)	460 (270–1380)	810 (430–2020)	490 (390–700)	1420 (910–2120)	0.17

Median (interquartile range) are shown. A decrease in HOMA-S and ISI is indicative of a reduction in insulin sensitivity.

* Changes in insulin sensitivity in this study have been previously reported [12]

On logistic analysis, treatment (placebo vs. dexamethasone) was associated with change in insulin sensitivity, and remained after individual adjustment for age, and changes in BMI, blood pressure, and HDL cholesterol: β = -3.39, *P *< 0.001 (as reported with HOMA-S [12]). Using partial correlation analysis (adjusting for treatment arm), associations between the change in each measured variable with changes in insulin sensitivity were investigated. Of variables analysed (including blood pressure, BMI and fasting metabolic indices: serum lipids, non-esterified fatty acids, TNF alpha, adiponectin, leptin, ghrelin), only systolic blood pressure (partial correlation coefficient: -0.50, *P *= 0.01) and diastolic blood pressure (-0.48, *P *= 0.02) were associated with HOMA-S and ISI.

## Discussion

Contrary to our hypothesis, short-term dexamethasone treatment did not significantly change levels of TNF alpha, ghrelin, leptin or adiponectin, despite a treatment-related hyperinsulinaemic response [12]. The implication is that GC-mediated insulin resistance does not result from nor elicit major changes in these metabolically active hormones. Data here pertain only to insulin resistance associated with short-term exogenous glucocorticoid treatment, since other etiologies of insulin resistance may result from fundamentally different mechanisms.

Dexamethasone-induced insulin resistance remains a complex mechanism [15] that is suggested to involve changes in whole body free fatty acid turnover, plasma insulin concentrations [16] and alterations in both insulin signal transduction [17] and glucose transporters [18] Both leptin and adiponectin promote catabolic energy generating processes, such as the mobilisation of triglycerides stores to promote fatty acid oxidation [19]. In line with our earlier report of a lack of effect on fasting NEFA levels [12], data here argue against a role of aberrant NEFA regulation as a mechanism of glucocorticoid-induced insulin resistance. Also, while whole body lipolysis is different between men and women [20] there is no gender variation in dexamethasone induced insulin resistance [16]. Hence, this disordered NEFA metabolism reported with dexamethasone-induced insulin resistance may be consequential of changes involving signal transduction and glucose transport.

Circulating levels of TNF alpha show a coordinated increase with obesity during the course of gestational diabetes [21], and at a physiological level, this adipocytokine alters insulin signal transduction [22] and secretion [23]. Moreover, adiposity correlates with plasma levels of pro-inflammatory cytokines such as TNF alpha and the systemic acute phase protein C-reactive protein (CRP). In this study we have already reported that dexamethasone therapy resulted in a decrease in CRP levels [12]. Circulating CRP levels are suggested to relate to adipose derived mediators such as leptin and TNFα, and positively correlate with measures of obesity in otherwise healthy adults [24,25]. In the present analysis we found no association between absolute levels or dexamethasone-related changes in CRP with metabolically active hormone levels.

It is important to place our findings in the context of other studies examining the impact of glucocorticoids on metabolically active hormones and cytokines. Specifically, some human studies suggest that glucocorticoids may decrease ghrelin levels [26] and increase leptin levels [27]. However, one group reported that fasting obliterated the increase in leptin in response to exogenous glucocorticoids [28] which may account for the lack of a rise in leptin concentrations with glucocorticoid treatment in our subjects. In longer-term studies, the impact of glucocorticoids on metabolically active cytokines and hormones may be mediated by changes that accompany more chronic glucocorticoid effects, such as obesity [29], rather than by direct glucocorticoid effects. While it is conceivable that a larger sample size, longer treatment duration, or non-fasting blood assays might have generated positive findings, the highly significant change in insulin sensitivity we observed with dexamethasone reassures us that our study design allowed for detection of major alterations in metabolic cytokines and hormones. Furthermore, the since all subjects who received dexamethasone had undetectable post-treatment cortisol levels, we know that our negative findings were not a result of noncompliance with the intervention. Based on these factors, we suspect that any effects of short-term glucocorticoids on circulating levels of metabolic cytokines and adipokines are likely small, if indeed present at all.

In summary, this randomised placebo-controlled study provides insight into the effects of glucocorticoids, without interference from pathological disease states that are commonly manifest amongst patients on GC therapy. Short-term dexamethasone therapy did not significantly change circulating concentrations of metabolically active hormones, despite increasing insulin resistance.

## Competing interests

The author(s) declare that they have no competing interests.

## Authors' contributions

JVP, DEC and AVM carried out the hormone assays, and drafted the manuscript. DJB participated in the design of the study and assisted the statistical analysis (with JVP) and conceived of the study, GYHL, JPG, MG and SR also participated in the study design and its coordination/recruitment of subjects. EAH, DJB, JPG and SR contributed to the funding of this research.
